# Feeding a *Saccharomyces cerevisiae* Fermentation Product to Mares in Late Gestation Alters the Biological Activity of Colostrum

**DOI:** 10.3390/ani14172459

**Published:** 2024-08-24

**Authors:** Eva Ronja Terpeluk, Jana Schäfer, Christa Finkler-Schade, Elke Rauch, Karl Rohn, Hans-Joachim Schuberth

**Affiliations:** 1 Institute for Immunology, University of Veterinary Medicine Foundation, Bünteweg 2, 30559 Hannover, Germany; eva.terpeluk@tiho-hannover.de; 2 Schäfer Horse Breeding, 38159 Vechelde, Germany; jana_schaefer@outlook.com; 3 Schade & Partner, 27283 Verden, Germany; cs@schadeundpartner.de; 4 Chair of Animal Welfare, Ethology, Animal Hygiene and Animal Husbandry, Department of Veterinary Sciences, Faculty of Veterinary Medicine, LMU Munich, 80539 Munich, Germany; 5 Institute for Biometry, Epidemiology and Information Processing, University of Veterinary Medicine Foundation, Bünteweg 2, 30559 Hannover, Germany

**Keywords:** colostrum, biological activity, horses, foals, *Saccharomyces cerevisiae*, SCFP

## Abstract

**Simple Summary:**

To ensure adequate immune protection, foals are dependent on a sufficient intake of good quality colostrum shortly after birth. In this study we analyzed whether a supplemented *Saccharomyces cerevisiae* fermentation product (SCFP) to mares in late gestation alters the biological activity of their colostrum and whether the uptake of such a colostrum affects the immediate response to vaccination at the age of 5 to 8 months. Twenty-six pregnant mares were randomly assigned to receive either a supplement or no supplementation twelve weeks before the calculated date of birth. Mares were actively vaccinated three weeks before parturition with a commercial influenza and tetanus vaccine. Their foals were actively vaccinated against equine influenza and tetanus after weaning. Blood cell composition was determined directly before vaccination and 24 h later. The biological activity of colostrum samples was assessed in a cell viability assay with an intestinal epithelial cell line. Colostrum from SCFP-fed mares exhibited significantly higher growth-promoting activity compared to control mares. While SCFP feeding did not affect the early vaccination response of mares, the biological activity of colostrum had a significant effect on vaccine-induced changes in blood composition of weanling foals. Therefore, a *Saccharomyces cerevisiae* fermentation product supplement may improve the biological activity of colostrum, which affects innate immune responses of foals.

**Abstract:**

The quality of equine colostrum is typically defined by refractometry or the concentration of maternal antibodies. However, the activity of other equine colostral bioactive molecules has not yet been investigated. This study analyzed whether the administration of a *Saccharomyces cerevisiae* fermentation product (SCFP) influences the biological activity of mare colostrum and whether the biological activity of colostrum has a lasting immunomodulating effect for foals. A total of fourteen pregnant mares received 20 g/day of a SCFP for a period of twelve weeks prior to the calculated date of birth (SCFP-group). Twelve pregnant mares without supplementation served as controls (CON). Colostral Brix values were determined within three hours after parturition. The concentration of IgG in blood sera and colostrum samples was determined with an ELISA. The biological activity was determined in a cell growth assay with a porcine epithelial cell line (IPEC-J2). Mares (at three weeks before the calculated date of birth) and foals (at the age of 5–8 months) received a parenteral active vaccination against influenza and tetanus. The administration of SCFP did not alter the mare’s serum and colostrum IgG concentrations and did not exert a significant influence on the mares’ early response to the vaccination. Growth and diarrhea episodes were comparable between foals of supplemented mares (SCFP) and foals of mares without supplementation (CON). Colostrum samples from SCFP-supplemented mares exhibited heightened biological activity. While SFCP and CON foals did not differ in their early response to vaccination, the vaccination-induced alterations in circulating neutrophilic granulocyte numbers were significantly correlated with the biological colostrum activity. These findings suggest that the supplementation of mares in late gestation with SCFP can enhance the biological activity of colostrum, which subsequently influences the innate immune responses of their offspring in later life.

## 1. Introduction

Colostrum, as the first source of nutrition for newborn mammals, plays a major role in the protection of health and the development of the immune system [1]. In addition to its protective function against environmental opportunistic pathogens, colostrum participates in the development of the neonatal immune system through a variety of bioactive factors [2]. However, the focus in equines is still on colostral antibody levels, as protection against pathogens in near agammaglobulinemic foals is mainly mediated by maternal antibodies [3,4]. Immunoglobulin G (IgG) is the dominant antibody isotype in equine colostrum. IgA, IgM and IgE are present in much lower concentrations [5]. Colostral IgG concentrations in horses can vary over a wide range. Colostral IgG levels below 28 g/L are considered inadequate, whereas adequate quality colostrum contains between 50 and 80 g/L [6]. Colostrum quality is more easily assessed by refracotmetry [5,6,7,8]. Brix values > 23% indicate good quality colostrum [5]. The factors influencing colostrum IgG content or %Brix are not completely clear. Some studies have shown no significant effect of age, parity, number of barren seasons, assisted delivery, retained placenta, or month of parturition on colostral or serum IgG concentrations in foals [9,10,11,12,13]. Other studies have identified parity, age, gestational age, and month of parturition as influencing factors [14,15,16,17]. In addition, mares’ serum IgG concentrations before and after parturition did not correlate significantly with colostral IgG concentrations [9,11,14,18,19].

In addition to immunoglobulins, colostrum contains a variety of other biologically active molecules, such as growth factors, cytokines, chemokines, complement components, exosomes, and oligosaccharides [2,20,21,22,23,24,25]. Their relative importance for neonatal development and the immune system has been the subject of several reviews on human, bovine, caprine, and porcine colostrum [3,7,8,20,26,27,28]. Many colostral molecules act as potent immune response modulators (colostral bioactives) [2], and the interaction of these molecules with gut intestinal cells also determines the IgG uptake capacity of neonates. However, serum IgG concentrations of newborn foals after colostrum ingestion correlate weakly (r ≤ 0.462) with the colostral IgG content of the dam [12,19,29].

The assessment of colostrum bioactivity is not trivial and is currently performed in vitro using human and porcine intestinal epithelial cell lines [1,21,30,31,32,33]. By definition, the cell line growth-promoting effect of a colostrum sample describes its bioactivity. In vivo studies showed that only feeding a highly bioactive bovine colostrum sample inhibited the development of NSAID-induced gastric ulcers in rats [34]. Whether the bioactivity of colostrum, as determined with intestinal epithelial cells, also has long-term effects on other cell or immune responses is currently largely unknown. At least in cattle, simple measures such as freezing/thawing, which destroys viable maternal colostrum cells, had an effect on the immune response of calves up to 10 months of age [35], suggesting that feeding of colostrum with different biological activities has a long-term effect on the development of the neonatal immune system.

Nutritional attempts to increase colostral IgG levels and/or enteral uptake of colostrum included feeding *β*-glucans to mares, which significantly increased IgG concentrations in equine colostrum (74.14 ± 15.25 g/L) compared to the control group (53.80 ± 10.95 g/L) [7]. Feeding live *Saccharomyces cerevisiae* (*S. cerevisiae*) in the same experiment showed no significant effect on colostral IgG concentrations but resulted in significantly higher IgG concentrations in foal serum [7]. Ewes supplemented with live yeast (*S. cerevisiae*) showed higher colostral IgG levels [27]. In sows, supplementation with a *Saccharomyces cerevisiae* fermentation product (SCFP) improved lactation performance but not colostrum composition [36]. Other studies in sows showed either no effect of *S. cerevisiae* supplementation on colostral immunoglobulin concentrations [37,38,39,40] or an enhancing effect [28]. SCFP is a complex mixture of bioactive compounds, such as proteins, peptides, and metabolites, produced through the fermentation of the yeast *Saccharomyces cerevisiae*, commonly used to improve nutrition, digestion, health, performance and immunity in humans and multiple animal species [36,41,42,43,44,45]. Since feeding SCFP has an immunomodulatory effect in horses and foals [46,47,48], we hypothesized that feeding SCFP to pregnant mares would affect the biological activity of their colostrum. Therefore, we used a bioactivity assay for equine colostrum and analyzed whether the ingestion of colostrum with different bioactivities leads to an altered development of the immune system in foals, which is reflected in an altered response to vaccination later in life.

## 2. Materials and Methods

### 2.1. Animals and Supplementation

The study was conducted on a farm in Lower Saxony, Germany and approved by the State of Lower Saxony, Germany, in accordance with § 8(1) of the Animal Protection Act in conjunction with § 33 of the Animal Protection Experimental Animal Regulations (File number: 33.19-42502-04-22-00254). Twenty-six mares (approximately 500–700 kg body weight), Hannoveraner breed, in gestation were assigned to two groups, SCFP-supplemented group (SCFP) and control group (CON), according to age and parity (Table S1). Their foals were classified accordingly with 4 fillies and 10 colts in the group of supplemented mares (SCFP) and 5 fillies and 7 colts in the control group (CON). All foals were born in the 2023 foaling season with the first foal born in mid-January and the last two in early June. The treatment groups were balanced for expecting foaling date (Table S2). All mares were housed in the same facility in individual stalls. They were handled in the same manner with respect to food, feeding times, and turnout times. Their foals were treated as similarly as possible. Depending on weather conditions, they had access to different areas (pasture, paddock, indoor arena). Horses (SCFP and CON) were comingled during turnout before parturition, the first 30 days after parturition as well as after the 30-day observation period on permanent pastures. Due to different birth months, it was not possible to provide identical turnout handling for all foals. During the first 30 days, the group size was variable and depended on the area (for example: max. 4 mares with their foals in the indoor arena, max. 6 mares and their foals on the pasture) and on the number of foals born. After the 30 days period, groups of 6–16 mares with their foals were moved to permanent pasture, depending on pasture size and amount of grass, regardless of previous supplementation classification.

**Figure 1:**
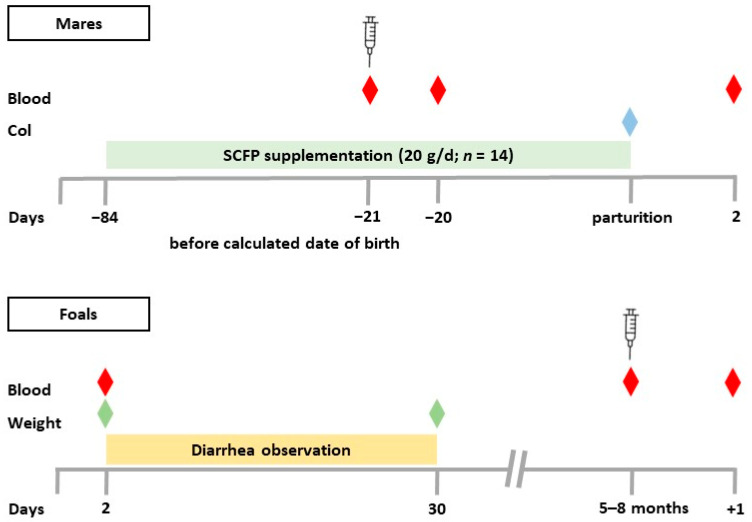
Experimental design. Twenty-six pregnant mares were randomly assigned to two groups. The supplemented group (SCFP, *n* = 14) received 20 g of the SCFP daily beginning 12 weeks before the expected date of birth. The control group (CON, *n* = 12) received no supplementation. Blood samples (red diamond) were collected from the mares on the day of vaccination (3 weeks before the expected date of birth), 24 h after vaccination (−20), and the day after parturition. Colostrum (Col, blue diamond) samples were collected within 3 h of delivery. Diarrhea incidence in the foals was determined daily between day 2 and day 30. Body weight (green diamond) was measured on days 2 and 30. Blood (red diamond) samples were collected from the foals on day 2, on the day of vaccination (age 5–8 months), and 24 h after vaccination (+1); calculated date of birth = last date of mating +11 months.

The mares’ diet consisted of high-quality hay, barley, oats and minerals in approximately equal amounts twice daily. They were fed in their individual stalls in the morning at 6.30 am and around 4.30 pm in the afternoon. Hay was produced in-house by the farm from first-cut green forage. Mares in late gestation received 8 kg per day and in lactation 10 kg per day divided into morning and evening portion. Oats and barley also came from the farm’s own production. In late gestation, mares received 1.5 kg oats and 1.5 kg barley per day, in lactation 2.5 kg/d each. During lactation, 0.5 kg soybean meal (Agravis Raiffeisen Markt, Vechelde, Germany) was added to the daily ration. The diet included 100 g of minerals (7 mineral bricks, Heinrich Eggersmann Futtermittelwerke GmbH, Extertal, Germany) per day. Foals were fed hay, oats, barley and minerals in equal amounts after weaning. In addition, mares and foals had access to the bedding material (wheat straw). During their stay on the permanent pasture, no concentrated feed was fed, but grass and mineral licks were freely available. Mares and foals had access to water at all times. The supplement was administered to mares (*n* = 14) with the concentrate during the morning feeding, starting 12 weeks before the calculated date of parturition and ending at parturition (Figure 1). The supplementation for the first mare started mid-November 2022 and for the last mare end of March 2023. They received 20 g dry powder of the SCFP according to the recommendation of the supplier and the successfully conducted, previous study [46]. The feeding trough was checked for complete supplement and concentrate intake. All mares showed good supplement intake without any feed refusal. The control group (CON, *n* = 12) received no supplementation. Table 1 lists the ingredients of the used SCFP Olimond BB (BB horses GmbH & Co. KG, Kreuztal, Germany). All mares were fed the supplement from one single batch for the entire study. The composition of the supplement is listed in Table 1. Analysis of the nutrient content was reported by the company. It is available in all EU member states.

One SCFP mare died after parturition, and the associated foal died a few days later. The mare’s previously collected vaccination data were not evaluated. One SCFP foal had to be euthanized during the study due to an arthritic fetlock joint. Foal #3 (SCFP) received a 6-day course of trimethoprim-sulfamethoxazole (TMS) for pulmonary infection on day 48 postpartum. Mare #4 (CON) was pre-treated with ceftiofur approximately 10 days prior to foaling. On day 53, foal #4 (CON) suffered a right rib trauma due to kicking by the mare, no ventilation, mild epistaxis and was treated with meloxicam and TMS for 7 days. Foal number 7 (SCFP) was stapled for entropion on the right side. Foal number 19 (CON) had a corneal defect 2 months after birth and was treated with antibiotic ointment (ofloxacin). Foal number 21 (SCFP) was given meloxicam on day 47 for 5 days due to lameness. Foal number 25 (SCFP) was treated for *Rhodococcus equi* 3 months after birth.

The following deworming regimen was applied to the mares: Ivermectin (Bimectin Paste 18.7 mg/g, Bimeda^®^, Dublin, Ireland) on the day of foaling, Pyrantel (152.2 mg/g, CP-Pharma Handelsgesellschaft mbH, Burgdorf, Germany) groupwise in spring regardless of parturition stage, and Moxidectin + Praziquantel (Equest^®^ Pramox 19.5 mg/g + 121.7 mg/g, Zoetis Deutschland GmbH, Berlin, Germany) groupwise in fall after weaning. Foals were dewormed with Pyrantel at six weeks of age, which was repeated every 6 weeks. After weaning and active vaccination, Ivermectin was administered to the foals. Information about active vaccination can be found below (Section 2.6).

### 2.2. Weight Determination and Diarrhea Observation

Foals were weighed on days 2 and 30 (floor scale, SBS-PS-300, Steinberg^®^ Systems, Berlin, Germany) (Figure 1). The diarrhea occurrence was specifically documented during the morning routine by the farm veterinarian for a period of 29 days, starting on day 2. Feces with a watery or thin mushy consistency were classified as diarrhea (Table S3). The severity of diarrhea was not assessed.

### 2.3. Colostrum and Blood Sample Collection

Colostrum specimens were collected immediately after the foal was born and before the foal’s first suckling. The colostrum refractive index was determined with a Brix refractometer (RHB-190OE, HHTEC^®^, Hong Han GmbH, Heidelberg, Germany). For the collection of colostrum, a sterile 50 mL centrifuge tube (SARSTEDT AG & Co. KG, Nümbrecht, Germany) was used. After discarding the first colostral streams from both sides, the tube was filled with 40–50 mL colostrum except for the sample of mare number 4 with approximately 20 mL because this mare hardly gave any milk. The colostrum intake of the foals was also monitored. As mare number 4 was barely giving milk, the foal was bottle-fed with colostrum (frozen from two mares in 2022) and commercial foal milk. The mare #4 was part of the control group, so the foal was not excluded from this study.

Since the foal #4 received foreign colostrum, the bioactivity was determined in the mixture of the frozen/thawed colostral portions. Foal number 19 showed no suckling reflex. Therefore, colostrum was administered through a probe until the foal drank independently from the udder. The first colostrum intake of all foals was within 3 h of birth. Immediately before collecting the blood samples, animals were clinically examined (examination of lymph nodes, oral mucosa and the conjunctiva) and the rectal body temperature was measured with a clinical thermometer (SCALA Electronic GmbH, Stahnsdorf, Germany). Blood samples from mares were collected on the day of vaccination (serum and heparin), approximately 3 weeks before the calculated date of birth, 24 h after vaccination (heparin), and the day after parturition (day 2; serum).

Foal blood samples were taken on day 2 (serum), after weaning immediately before vaccination at 5–8 months of age (heparin and serum), and 24 h after vaccination (heparin) (Figure 1). Day 2 foal blood was collected by venipuncture (jugular vein) using a disposable needle (20 G × 1½, Sterican^®^, B. Braun SE, Melsungen, Germany) and a 10 mL syringe (Injekt^®^, B. Braun SE, Melsungen, Germany). Blood samples from mares and foals at 5–8 months of age were collected from the jugular vein into BD Vacutainer^®^ Sodium Heparin Tubes (Medicalis Medizintechnologie, Hannover, Germany) and BD Vacutainer^®^ Serum Tubes (Medicalis Medizintechnologie, Hannover, Germany). Day 2 blood from mares and foals was used to determine the total serum protein (TSP) with a refractometer (RHC-300, HHTEC^®^, Hong Han GmbH, Heidelberg, Germany) and processed for freezing as described below. Due to TSP values below 5.5 g/dL, a rapid IgG concentration test (Fassisi Equine IgG-test, Göttingen, Germany) was performed on 5 foals. Two foals from unsupplemented mares received a 2 L plasma transfusion. Serum tubes were centrifuged for 10 min with 3000 revolutions per minute to obtain clot-free serum. Serum was transferred to several 1.5 mL Eppendorf tubes (SARSTEDT AG & Co. KG, Nümbrecht, Germany) and, like the native colostrum samples, stored frozen at −20 °C until analysis. Analysis of viable blood cells was performed within 24 h of blood collection.

### 2.4. Determination Leukocyte Subpopulations

The total leukocyte count, and flow cytometric determination of the major leukocyte subpopulations (granulocytes, monocytes, and lymphocytes) have been described in detail [48]. Briefly, after hypotonic lysis of heparinized blood, lymphoid cells, granulocytes, and monocytes were identified based on their scatter characteristics after flow cytometry (BD Accuri™ C6 Flow Cytometer, Becton Dickinson Inc., Holdrege, NE, USA). To determine the total number of leukocyte subpopulations, their relative numbers were multiplied by the total leukocyte count. Due to previous technical problems with hypotonic lysis of foal blood, density separation was used in this study. Three mL of heparinized blood was diluted with three mL of phosphate-buffered saline (PBS) containing 0.02% ethylenediaminetetraacetic acid (EDTA). Percoll^®^ (Sigma-Aldrich Chemie GmbH, Taufkirchen, Germany) was diluted to 70% with PBS containing 0.02% EDTA. Six mL of 70% Percoll^®^ was added to a sterile fifteen mL centrifuge tube (nerbe plus GmbH & Co. KG, Winsen/Luhe, Germany). After slowly adding 6 mL of diluted foal blood, the tube was centrifuged (800× *g*, 30 min). One mL interphase was transferred to a four mL polystyrene tube (SARSTEDT AG & Co. KG, Nümbrecht, Germany) and one mL PBS containing 0.02% EDTA was added. After two washes (centrifugation at 500× *g* and 250× *g* for 10 min), the cell pellet was resuspended in 2 mL PBS containing 0.02% EDTA. One hundred µL was removed and transferred to a 1.5 mL Eppendorf tube (SARSTEDT AG & Co. KG, Nümbrecht, Germany) and one hundred µL sterile-filtered PBS (0.4 μg/mL propidium iodide) was added. Flow cytometric analysis was performed as described [48].

### 2.5. Membrane Immunofluorescence

Leukocytes were incubated with horse-specific and horse-cross-reactive monoclonal antibodies as previously described [48]. Briefly, leukocytes were incubated in three separate sets with monoclonal antibody (mAb) combinations (30 μL) for 30 min on ice. Used antibodies were specific for equine CD4, equine CD8, canine CD21 (cross-reactive with horse cells), and equine MHC class II. Details of antibody supplier, isotypes and used dilutions are mentioned in Terpeluk et al. [48]. After washing, cells were analyzed by flow cytometer. After gating for viable and single cells [40], the fractions of CD4+ T cells, CD8+ T cells, CD21+ B cells, and MHC-II+/CD21- lymphoid cells were determined and the total number of lymphoid cells/mL was calculated. Fluorescence compensations were performed post-acquisition using the BD Acurri C6 plus software version 1.0.34.1 [42]. For foal blood, the mAb combinations were slightly changed for set 1: anti-eqCD4-FITC, anti-eqCD8-RPE, and anti-canine CD21-AlexaFluor^®^647. Sets 2 and 3 [48] remained the same.

### 2.6. Vaccination

Mares were actively vaccinated on average 21.5 ± 5.1 days before the calculated date of birth. Foals were actively vaccinated after weaning at 5–8 months of age. To ensure that all animals were in good health, they were clinically examined, and rectal body temperature was measured before vaccination. A commercial influenza and tetanus vaccine was used for mares and their foals (PROTEQFLU™-TE; 1 mL Influenza A/eq/Ohio/03 [H3N8] recombinant of canarypox virus (strain vCP2242), Influenza A/eq/Richmond/1/07 [H3N8] recombinant of canarypox virus (strain vCP3011) and Clostridium tetani toxoid ≥ 30 I. U. with carbomer as adjuvant, Boehringer-Ingelheim Vetmedica GmbH, Ingelheim am Rhein, Germany). Following standard cleaning, the vaccine was injected intramuscularly (left pectoral muscle) with a disposable needle (22G × 1¼, Sterican^®^, B. Braun SE, Melsungen, Germany) and a 3 mL syringe (Omnifix^®^ Luer Solo, B. Braun SE, Melsungen, Germany). Twenty-four hours after injection, mares and foals were reassessed for vaccine side effects, including swelling at the injection site, fever, pain or a decline in overall condition. Three mares responded with a soft, fluctuating swelling without treatment required. None of the mares had an elevated rectal temperature either before or 24 h after vaccination. One foal (SCFP) reacted with elevated temperature 24 h after vaccination and was treated after blood sample collection with meloxicam, metamizole and flunixin. Another foal (SCFP) showed mild colic symptoms a few hours after vaccination without medical treatment required.

### 2.7. Determination of Immunoglobulin G

Immunoglobulin G (IgG) was determined by sandwich enzyme-linked immunosorbent assay (ELISA). Colostrum samples, serum samples from mares the day of vaccination (3 weeks before the calculated date of delivery), day 2 serum samples from mares and foals and foals’ serum samples the day of vaccination (at the age of 5–8 months) were analyzed. 96-well ELISA plates (Maxisorb, Nunc^®^, Wiesbaden, Germany) were coated with rabbit anti-horse IgG antibody (Cat. No: SAB3700150, 0.5 µg/mL, Sigma-Aldrich Chemie GmbH, Taufkirchen, Germany, 100 µL/well) and incubated for 18 h at 4 °C. After three washes with PBS-Tween (PBS-T) using a mechanical washer (ELx405, BIO-TEK Instruments GmbH, Bad Friedrichshall, Germany), 200 µL/well 0.5% gelatin solution (Serva GmbH, Heidelberg, Germany) was added (1 h, 37 °C) to block vacant binding sites. After washing the wells once, postcolostral foal serum (day 2) was diluted with PBS-T (1:5000), and the remaining samples (foal sera (5–8 months), mare sera and colostrum samples) were diluted 1:50,000. Diluted samples were added to the top wells and a 2-logarithmic dilution series was established in each column resulting in a final loading of 50 µL/well. Column 1 served as a blank and was filled with 50 µL PBS-T per well. Equine IgG was used as a standard at an initial concentration of 1.0 µg/mL and diluted in a two logarithmic series. After incubation for 1 h at 37 °C, a wash step was added. Subsequently, 100 µL/well of 1:20,000 diluted rabbit anti-horse IgG peroxidase (article number: A6917, Sigma-Aldrich Chemie GmbH, Taufkirchen, Germany) was added and incubated for 1 h at 37 °C. After a washing step, 100 µL of tetramethylbenzidine (TMB) solution (10 mL TMB buffer + 322 µL TMB stock + 3 µL H_2_O_2_) was added to each well and incubated for 10 min at room temperature in the dark. The peroxidase splits H_2_O_2_ into H_2_O and O_2_, and the substrate is converted, resulting in a blue color. Fifty µL/well H_2_SO_4_ was used to stop the enzymatic reaction and change the blue color to yellow. The plates were read photometrically using an ELISA reader (Synergy HT, BIO-TEK Instruments GmbH, Bad Friedrichshall, Germany) at 450 nm. The IgG concentrations of the samples were calculated using the standard curve (Gen5 software version 3.16.10, BIO-TEK Instruments GmbH, Bad Friedrichshall, Germany). The standard curve (8 points per curve) ranged from 17 to 1000 ng IgG/mL.

### 2.8. In Vitro Determination of the Biological Activity of Colostrum

The RealTime-Glo™ (RTG) assay (Promega, Walldorf, Germany) and porcine intestinal epithelial cells (IPEC-J2) (Leibniz Institute DSMZ-Deutsche Sammlung von Mikroorganismen und Zellkulturen GmbH, Braunschweig, Germany) were used to determine the biological activity of colostrum. The RTG assay is a cell viability assay that generates a luminescent signal and consists of two solutions: MT Cell Viability Substrate and NanoLuc^®^ Luciferase. The MT Cell Viability Substrate diffuses into cells where it is reduced to form a NanoLuc^®^ substrate, which exits the cell and is immediately utilized by the NanoLuc^®^ Luciferase. Light production is proportional to the number of viable cells in the culture. Dead cells are unable to reduce the substrate. The IPEC-J2 cell line was isolated from the jejunum of a newborn piglet and is not transformed. Cells were cultured in Dulbecco’s modified Eagle’s medium/F-12 Ham (DMEM/Ham) (Sigma-Aldrich Chemie GmbH, Taufkirchen, Germany) supplemented with 10% fetal calf serum (FCS) and 1% penicillin-streptomycin (PS) (PAN-Biotech GmbH, Aidenbach, Germany) in two culture flasks (T-175, filter cap, SARSTEDT AG & Co. KG, Nümbrecht, Germany) in an incubator (Heracell 240, series 5060; Heraeus Medevio, Hanau, Germany) at 37 °C, 5% CO_2_.

Based on preliminary experiments, a final colostrum concentration of 1:50 and an incubation time of 24 h were used. Colostrum samples were pre-diluted 1:25 with PBS and sterile filtered (0.2 µm, syringe filter, Filtropur S, SARSTEDT AG & Co. KG, Nümbrecht, Germany). Cells were harvested by 20 min incubation with 3 mL Accutase (Sigma-Aldrich Chemie GmbH, Taufkirchen, Germany) per culture flask. After addition of culture medium DMEM/Ham containing 10% FCS and 1% PS, the cell mixture was transferred to a 50 mL falcon. Two mL cell mixture was left in the culture flask and supplemented with the described culture medium. Cells were washed twice at 500× *g* for 10 min, and the second time with serum-free culture medium containing 1% PS (SFM). Cells were resuspended in a defined volume of SFM. Cells were then counted using a Bürker chamber and adjusted to 400 cells/well. The two RTG solutions were added to the adjusted cells (2 µL per solution for 1 mL of cells). SFM and SFM containing 1% FCS were used as controls. Twenty µL/well of the controls and the 1:25 pre-diluted colostrum samples were pipetted into a 384-well plate (flat bottom, white, polystyrene, Greiner Bio-One GmbH, Frickenhausen, Germany). For pipetting, a Multipette^®^ (M4, Eppendorf Vertrieb Deutschland GmbH, Hamburg, Germany) was combined with a Combitip^®^ advanced (1 mL, Eppendorf Vertrieb Deutschland GmbH, Hamburg, Germany). The final dilution of the colostrum samples after adding 20 µL/well of the adjusted cells containing RTG solutions was 1:50. Four wells per sample were used as pipetting controls. In addition, new control samples were added to each second row, and control and colostrum samples were added to the last row but filled with SFM containing RTG solutions without cells. To ensure approximately the same number of cells per well, the plate was measured immediately after pipetting using a microplate reader (Tecan Infinite^®^ M1000, Tecan Group Ltd., Männedorf, Switzerland). The next measurement was made after 24 h of incubation at 37 °C, 5% CO_2_. Four experiments were performed at different times under the same conditions. For more comparable results, the measured luminescence values were related to the respective 1% FCS control (% of 1% FCS) and the average was determined for each sample (4 wells).

### 2.9. Statistical Analysis

The data analysis was conducted using SAS^®^ statistical software, version 9.4M7 and SAS Studio Enterprise, version 3.8.2 (SAS Institute Inc., Cary, NC, USA). Data visualization was performed using GraphPad Prism v9.0.0 (for Windows 64-bit, GraphPad Software, San Diego, CA, USA). *n* expresses the animal count. For the blood samples, the Shapiro–Wilk test was used to test for normal distribution of residuals and assumed if *p* > 0.05. If normal distribution was rejected, the data were log transformed and tested again for normal distribution. If this was also rejected, the Wilcoxon two-sample test was used (mares: reticulocytes; foals: reticulocytes; neutrophil–lymphocyte ratio (NLR)). Under the assumption of a normal distribution, a mixed model was run with the treatment/control group, vaccination time and combination as fixed effects, with the blood parameter or the log transformed data as dependent variables. The animals’ numbering was used as subject for identification. Vaccination time was also entered as a repeated measure (same animal; dependent measure). For foals’ blood evaluation, the differences in the blood cell parameter were calculated (post vaccination-pre vaccination) and the IPEC mean values, %Brix or IgG values, were included in the described mixed model as a continuous variable. %Brix, TSP, diarrhea days and daily weight gain were examined using the Wilcoxon two-sample test. Weight data were determined using a mixed model with Tukey-Kramer adjustment after visual observation of the residual distribution, with weight as the dependent variable, groups (supplemented/control), day (2 and 30) and interaction as fixed effects, foal ID as a subject for identification, and day also as a repeated measure. The average of the IPEC 24 h values (% of 1% FCS) of the 4 experiments was calculated and then compared between groups (SCFP, CON) with an unpaired pooled *t*-test, assuming normal distribution (Shapiro–Wilk) and equality of variances (folded F-test). IgG levels (serum and colostrum) were tested with an unpaired pooled *t*-test after assuming normal distribution (Shapiro–Wilk) and checking equality of variances with a folded F-test. Pearson correlation was used for correlation analysis. *p*-values ≤ 0.05 were considered statistically significant, while *p*-values between 0.1 and 0.05 were classified as indicative of a trend.

## 3. Results

### 3.1. SCFP Supplementation Did Not Influence the Immediate Vaccination Response of Pregnant Mares

At the time of vaccination, the mares did not differ significantly in the number or composition of their leukocytes (Table 2). There were no significant differences in the levels of leukocyte subpopulations such as granulocytes, lymphocytes and monocytes. Twenty-four h after vaccination, no significant differences in leukocyte counts or leukocyte subpopulations were found between both groups. Compared to pre-vaccination, the number of circulating leukocytes, granulocytes and monocytes increased postvaccination in both groups. Numbers of lymphocytes, CD4^+^ T cells, CD8^+^ T cells, CD21^+^ B cells and MHCII^+^/CD21^−^ cells decreased after vaccination (negative difference; Table 2).

### 3.2. SCFP Feeding Did Not Significantly Alter Serum IgG Concentrations in Mares

The immunoglobulin G concentration in serum at the time of vaccination as well as in serum after parturition did not differ significantly between supplemented mares and mares of the control group (Table 3). The IgG concentration of mares’ serum at the time of vaccination correlated moderately with the IgG concentration of mares’ serum after parturition (*r* = 0.626).

### 3.3. Colostrum Brix Values and Antibody Levels in Colostrum and Serum of Foals

**Figure 2:**
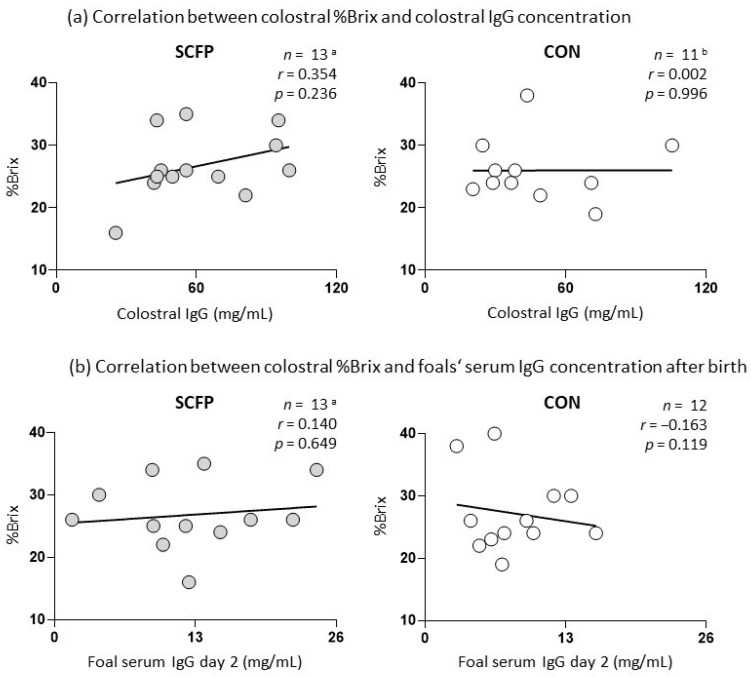
Correlation plots illustrating the correlation of colostral Brix values with colostral IgG and IgG concentration in foal serum after parturition (day 2); ^a^ one SCFP sample was excluded from the study due to death of a mare and foal shortly after foaling; ^b^ one CON colostrum sample was not usable for IgG detection; SCFP: supplemented group; CON: control group; *n*: number; *r*: *r*-value; and *p*: *p*-value.

The colostrum refractometric index of the supplemented group ranged from 16% to 35% (median 26%) and that of the control group from 19% to 40% (median 25%). The colostral IgG concentration of supplemented mares (61.4 ± 24.1 mg/mL) was higher than the colostral IgG concentration of the control group (47.3 ± 25.9 mg/mL) without statistical significance (*p* = 0.180). Foals born to SCFP-supplemented mares showed a trend towards higher postpartum IgG concentrations on day 2 (12.4 ± 6.4 mg/mL) compared to foals of the control group (8.3 ± 3.9 mg/mL, *p* = 0.072). IgG concentrations in foal serum did not correlate with IgG concentrations in colostrum (r = 0.049). No significant correlation was found between %Brix and IgG concentrations in colostrum or foal serum at the time of parturition (Figure 2).

### 3.4. SCFP Feeding Did Not Affect Foal Growth

Mares were divided into two groups according to age, parity and expected date of birth (Tables S1 and S2) to create equal conditions for the foals. Foals in both groups consumed colostrum of similar Brix quality (Table 4). The total serum protein after colostrum intake was comparable between foals of supplemented mares and foals of the control group (Table 4). Daily weight gain and final weight at day 30 were also similar in both groups (Table 4).

### 3.5. SCFP Feeding Altered the Biological Activity of Colostrum

**Figure 3:**
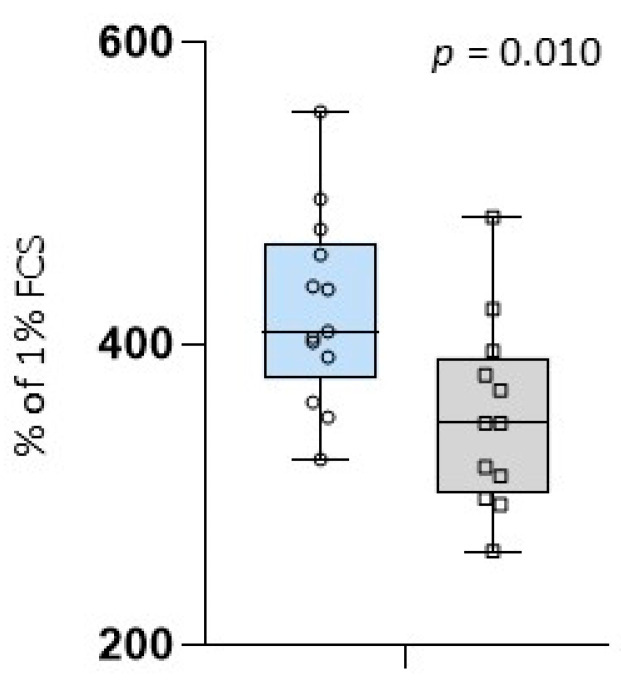
Average value of cell growth incubated in colostrum given in % of 1% FCS; blue box: colostrum samples of supplemented mares (SCFP); grey box: colostrum samples of the control group (CON); circles and squares show the individual values of each colostrum sample; and *p* = *p*-value.

The in vitro porcine epithelial cell line experiment showed significantly greater growth of IPEC-J2 cells incubated in colostrum from SCFP supplemented mares with an average of 423 ± 63.4% of 1% FCS compared to colostrum from the control group with an average of 352 ± 62.3% of 1% FCS (*p* = 0.010) (Figure 3). The colostral bioactivity did not correlate significantly with Brix values or IgG concentrations of colostrum and foal serum at day 2 (Table 5).

### 3.6. Biological Activity of Colostrum Affects Vaccination Response of Foals

**Figure 4:**
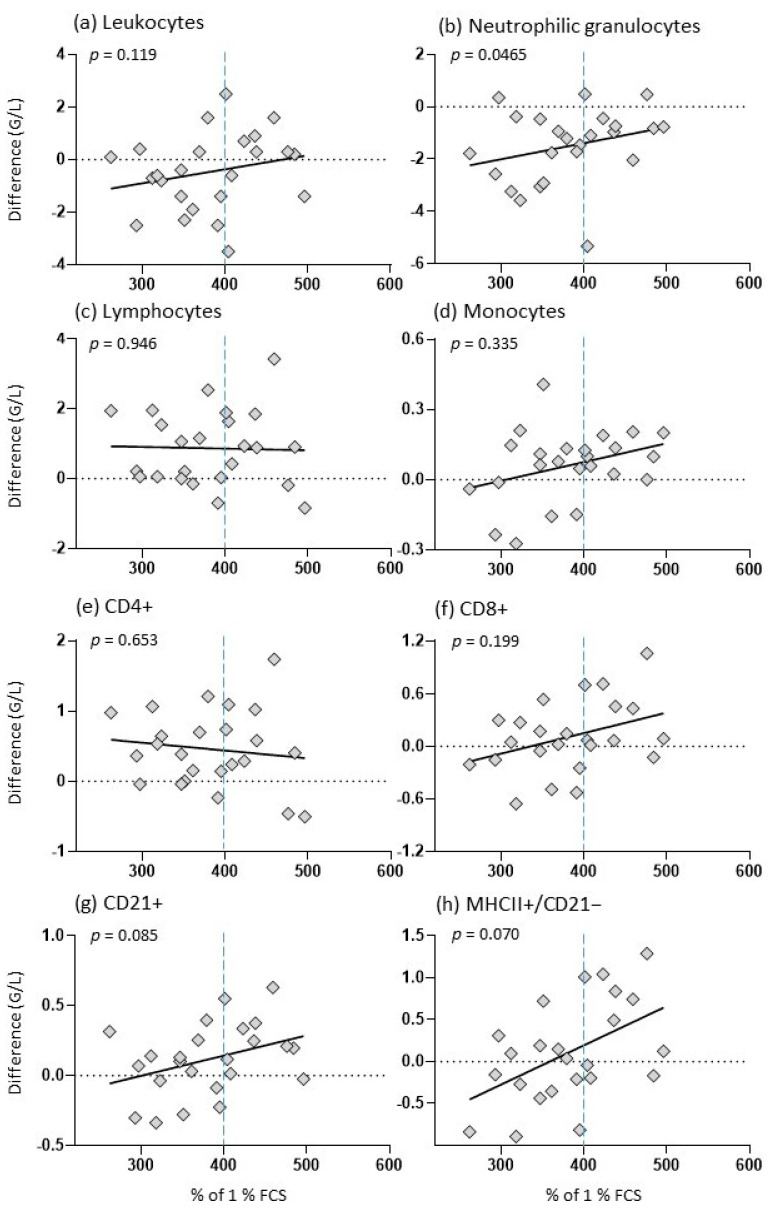
Vaccine-induced changes in foals in relation to colostral bioactivity. The *y*-axis indicates the vaccination difference (post-vaccination–pre-vaccination) and the *x*-axis indicates the colostral bioactivity in % of 1% FCS; the graph shows a dashed horizontal line at 0, a dashed vertical line at 400% and a regression line; the diamonds show the individual values of each foal; and *p*: *p*-value.

Vaccine-induced differences in the blood composition of foals at 5 to 8 months of age correlated significantly with the bioactivity of colostrum samples in terms of neutrophil granulocytes (Figure 4b). A trend was observed for CD21+ B cells and MHCII+/CD21− (Figure 4g,h). The mares producing colostrum with higher biological activity (above 400% of 1% FCS) tended to have foals with a larger difference in leukocytes, neutrophils, monocytes, CD8+ T cells, CD21+ B cells and circulating MHCII+/CD21− cells, whereas mares producing colostrum with a biological activity below this threshold tended to have foals with a smaller difference in cell numbers. While the foals’ vaccine-induced reaction of lymphocytes remained unchanged regarding the colostral bioactivity, foals which ingested colostrum with higher bioactivity tended to have smaller differences in CD4+ T cells compared to foals ingesting lower bioactive colostrum (Figure 4c,e).

The observed vaccination-induced changes in circulating blood cell counts were not correlated with %Brix, colostrum IgG content, or day 2 foal serum IgG concentration (Table S4).

## 4. Discussion

The feeding of *Saccharomyces cerevisiae* fermentation products has been demonstrated to exert immunomodulatory effects in a multitude of species. Prior research in horses has predominantly focused on the direct impact of SCFP on racehorses and foals [46,47,48], with long-term immunomodulatory outcomes being a key outcome measure. In newborn foals, the early supplementation of SCFP did not affect the frequency of diarrhea during the first three weeks of life. However, it was observed to influence the immune response to vaccination in later life stages [48]. The present study focused on the indirect effects of SCFP feeding on foals via the supplementation of pregnant mares with SCFP. The indirect effects of food additives on the offspring of pregnant mothers have been demonstrated in humans [20], horses [7], sows [28,36] and ewes [27].

The study focused on the potential impact of SCFP on colostrum quality, given the crucial role of colostrum in foal development [4,49]. However, the colostral Brix values of SCFP-fed mares were not significantly different from those of CON mares (*p* = 0.645, Table 5). In both groups, mean Brix values (25% and 26%) were comparable to those observed in a previous study involving live *S. cerevisiae*-fed (25%), β-glucan-fed (29.7%), and control mares (19.6%) [5]. Notably, the colostral IgG levels of SCFP and CON mares did not exhibit a significant correlation with the measured Brix values (Figure 2a). This is in contrast to the findings of Cash et al. [6] and Venner et al. [14], who demonstrated a strong correlation (*r* = 0.94 and *r* = 0.93, *p* < 0.01) between colostrum IgG levels and Brix values The discrepancy in the correlation may be attributed to the fact that the method of IgG quantification may have influenced the outcome. The unaltered Brix values and colostral IgG levels indicated that the feeding of SCFP to mares did not affect the quality of their colostrum. This was accompanied by unchanged IgG concentrations in the mares’ serum shortly after parturition. These findings are corroborated by those of Goncalves de Sobral et al. [7], wherein the administration of live *S. cerevisiae* (SC) to pregnant mares did not result in alterations to the serum IgG concentrations of mares postpartum, the colostrum Brix value, or the colostral IgG concentrations. However, significantly higher IgG levels were observed in colostrum samples of mares fed with pure *β*-glucan, a cell-wall component of SC, in comparison to the non-supplemented control group [7]. It is postulated that a potentially insufficient concentration of immunomodulatory *β*-glucan in our SCFP may explain the observed lack of change in IgG concentrations in colostrum of SCFP mares.

The unaltered response to parenteral vaccination (Table 2) after SCFP supplementation of mares is in stark contrast to studies reporting an altered vaccination response after SCFP supplementation in broilers [45], beef steers [50], racehorses [46], and weaned foals [48]. To the best of our knowledge, studies addressing the interplay between SCFP supplementation and the immune response to vaccination in late pregnancy have yet to be conducted. The results of this study suggest that the early innate responses to vaccinations in late pregnancy are tightly controlled and not easily modified by nutritional interventions.

Despite no significant difference in colostral Brix values and colostral IgG concentrations between SCFP and CON mares, foals from SCFP mares exhibited a tendency towards higher serum IgG concentrations compared to the control group (Table 4). This suggests that these foals have an enhanced capacity for IgG uptake, which is consistent with the findings of another study where foals born to mares fed live *S. cerevisiae* had significantly higher IgG serum concentrations compared to the control group [7]. Similarly, piglets of live yeast-supplemented sows exhibited elevated plasma IgG concentrations 24 h postpartum [51]. It is unclear whether this apparent enhanced capacity to absorb colostral IgG in the gut of newborn foals is programmed in utero. In other species, it has been demonstrated that a multitude of epigenetically active triggers during pregnancy, including distinct nutrients, impact the functionality of newborn gut epithelial cells and their developing immune system. [27,41,45,50,52,53]. In this study, we sought to determine whether SCFP feeding affects the biological activity of colostrum. This is a rather broad term for effects mediated by a plethora of ingredients, including growth factors, cytokines, exosomes, oligosaccharides, and others [2,22,54]. To date, no assay has been described for the assessment of biological activity in equine colostrum. To this end, an assay originally developed for human and bovine colostrum was adapted for use with an immortalized intestinal porcine epithelial cell line [21,31,55]. The IPEC-J2 cell line was selected due to its documented cross-species applicability [1,21,33] and the unavailability of a comparable equine gut epithelial cell line at the time of analysis. During the process of establishing the test, we transitioned from the CellTiter-Glo^®^ (CTG) luminescent cell viability assay (Promega, Walldorf, Germany), which permitted only a single measurement at a pre-specified time point, to the RTG reagent, which enabled the collection of data at multiple time points [56,57,58,59,60,61].

In comparison to colostrum from CON mares, colostrum samples from SCFP-supplemented mares demonstrated a notable enhancement in the growth of IPEC-J2 cells. This suggests that the supplementation of pregnant mares with SCFP may have improved the biological activity of colostrum (Figure 3). A comparable dietary intervention effect on the biological activity of colostrum for IPEC-J2 cells has been demonstrated in sows [32]. The precise manner by which SCFP modulates the bioactivity of colostrum remains uncertain. As previously proposed, resorbed SCFP metabolites may reach different body compartments and alter the reactivity and secretion capability of resident cells [46,47,48]. Furthermore, the absorption of postbiotics may also result in long-term epigenetic alterations [52,62,63]. Such cellular effects may also encompass the synthesis and/or transport of assorted biologically active molecules by mammary epithelial cells throughout the process of colostrogenesis. It has been demonstrated that feeding β-glucans or *S. cerevisiae* resulted in an increase in IgG levels in colostrum [7,28], as well as an elevation in the concentration of lactose in colostrum and protein and dry matter in milk [64].

The uptake of colostrum from SCFP-fed mares did not affect the growth performance of foals during their first 30 days of life (Table 4). This indicates that different colostrum qualities do not affect the capacity of nutritional absorption. Furthermore, there was no indication that the different biological activities affected the early development of the foals, as evidenced by insignificant differences in the days with diarrheic episode between CON and SCFP foals. 

In contrast to the lack of early effects on foal development, the biological activity of mare colostrum exerted a significant influence on the vaccination-induced change in the numbers of circulating neutrophils (Figure 4b), and in tendency of CD21+ B cells (Figure 4g), and MHC-II+/CD21- cells (Figure 4h) at the age of 5 to 8 months. The observed modulation in circulating cell numbers following vaccination suggests the presence of a differentially induced spectrum and concentration of cytokines, chemokines and other mediators, which guide the migration behavior of immune cells to and from primary and secondary immune organs. Such immediate vaccination responses have been documented in human vaccine trials [65,66,67] and in horses [46,48]. 

The role of equine colostral bioactivity in immune reactivity of foals later in life is partially reflected in a study with calves, in which the feeding of colostrum with lower bioactivity (lack of viable maternal cells after freezing and thawing) affected the vaccination response of calves at the age of 6 to 10 months [35]. It is currently unclear whether the modified vaccination response of foals after uptake of colostrum with different bioactivities will result in different antigen-specific antibody titers or whether it indicates altered immune mechanisms and responses against pathogens. In contrast to the colostral bioactivity, neither the colostral Brix value nor the colostral IgG concentration had a significant impact on the vaccination-induced changes of blood cell numbers (Table S4). Therefore, it can be concluded that the long-term immune-modulating effect of colostral ingredients is unlikely to be determined by classical parameters of colostrum quality. The manner in which equine colostrum exerts its bioactivity, as determined with a porcine epithelial cell line in vitro, remains uncertain with regard to its translation into the observed immunomodulation effect in foals. Potential mechanisms may include induced epigenetic changes in various cell types and organs [68], as well as long-lasting effects on the interplay between gut microbiota and the immune system [69,70].

## 5. Conclusions

The findings of this study indicate that supplementation of mares in late gestation with a *Saccharomyces cerevisiae* fermentation product can improve colostral bioactivity. This extends the definition of a good quality colostrum. The correlation of colostral bioactivity but not antibody content or refractive index with vaccination-induced immune responses of foals later in life indicates long-term effects of bioactive colostrum ingredients in foals.

## Figures and Tables

**Table 1:** Ingredients of the SCFP and the mineral supplement in the daily dose.

Ingredient	SCFP ^a^ (20 g)	Mineral Bricks ^b^ (100 g)
Crude protein	21.10%	
Crude fat	1.90%	
Crude fiber	19.20%	
Crude ash	9.20%	
Sodium (labelled)	0.00%	
Tocopherol extract	0.090 g	0.200 g
Vitamin C	0.584 g	0.050 g
Minerals (calcium carbonate)	0.126 g	12.00 g
Inactivated yeasts	19.200 g	

^a^ Supplemented mares received 20 g/day of a *Saccharomyces cerevisiae* fermentation product. ^b^ Mares of the supplemented and the control groups received daily 100 g minerals in form of bricks.

**Table 2:** Blood cell number differences after vaccination of mares.

	SCFP (*n* = 13) ^b^			CON (*n* = 12)			
Cell Type	Pre	Post	Difference	Pre	Post	Difference	*p*
Leukocytes	9.12 ± 2.24	11.80 ± 2.69	2.68 ± 1.63	9.41 ± 2.13	12.56 ± 1.93	3.15 ± 2.91	0.512
Granulocytes	7.07 ± 1.87	10.15 ± 2.63	3.08 ± 1.64	7.46 ± 2.03	11.00 ± 1.97	3.54 ± 2.87	0.404
Monocytes	0.34 ± 0.16	0.42 ± 0.20	0.08 ± 0.12	0.36 ± 0.18	0.42 ± 0.15	0.06 ± 0.18	0.829 ^a^
Lymphocytes	1.71 ± 0.99	1.23 ± 0.56	−0.48 ± 0.68	1.59 ± 1.11	1.14 ± 1.00	−0.45 ± 0.66	0.766
CD4^+^ T cells	0.90 ± 0.49	0.63 ± 0.25	−0.27 ± 0.39	0.82 ± 0.63	0.60 ± 0.63	−0.23 ± 0.35	0.764
CD8^+^ T cells	0.52 ± 0.34	0.36 ± 0.19	−0.16 ± 0.27	0.52 ± 0.37	0.36 ± 0.32	−0.17 ± 0.21	0.966
CD21^+^ B cells	0.13 ± 0.10	0.09 ± 0.08	−0.03 ± 0.10	0.11 ± 0.07	0.07 ± 0.06	−0.03 ± 0.06	0.468 ^a^
MHCII^+^/CD21^−^ cells	0.17 ± 0.16	0.13 ± 0.09	−0.04 ± 0.16	0.21 ± 0.23	0.16 ± 0.16	−0.04 ± 0.10	0.752 ^a^

Blood cell counts (G/L, mean ± SD) were determined at the day of vaccination (Pre) and 24 h later (Post). The difference was calculated between Post and Pre values; ^a^ for statistical analysis, data were transformed to a natural logarithm; ^b^ one mare was excluded from the study due to death after foaling; SCFP-supplemented group; CON: control group; and *p*: *p*-value.

**Table 3:** IgG levels in serum of mares at the time of vaccination and parturition.

	Vaccination	Parturition
SCFP (*n* = 13) ^a^	13.6 ± 3.1	14.5 ± 2.5
CON (*n* = 12)	12.9 ± 2.4	12.3 ± 4.4
*p*	0.5172	0.1350

Mean ± standard deviation (mg/mL); ^a^ one mare was excluded of the study due to death after foaling; SCFP: supplemented group, CON: control group; and *p*: *p*-value.

**Table 4:** Colostrum quality, serum parameter of newborn foals and their growth characteristics.

		Group 1 (SCFP) (*n* = 13 ^f^)	Group 2 (CON) (*n* = 12)	*p*
Colostral %Brix ^a^		26 (16–35)	25 (19–40)	0.645
Serum protein (g/dL) ^a,b^		6.0 (4.6–6.6)	6.4 (5.6–7.2)	0.946
Serum IgG (mg/mL) ^c^		12.4 ± 6.4	8.3 ± 3.9	0.072
Diarrhea days ^d^		4 ± 6	3 ± 5.5	0.882
Daily weight gain ^d,e^		1.23 ± 0.3	1.17 ± 0.5	0.299
Weight (kg) ^d^	d2	60.4 ± 7.8	56.5 ± 8.1	0.847
	d30	98.5 ± 15.1	89.9 ± 14.5	0.298

^a^ Median value (minimum–maximum); ^b^ total serum protein was determined on day 2; ^c^ mean ± standard deviation; ^d^ median ± interquartile range (IQR); ^e^ calculated from the weight difference of day 30–day 2 divided by 28 days; ^f^ one mare and the associated foal died after birth; and *p*: *p*-value.

**Table 5:** Correlation of colostral bioactivity with %Brix and IgG concentrations.

	SCFP Group			CON Group		
	*n*	*r*	*p*	*n*	*r*	*p*
%Brix	13 ^a^	0.0036	0.991	12	−0.085	0.794
Colostral IgG	13 ^a^	−0.222	0.467	11 ^b^	−0.172	0.613
Foal serum IgG day 2	13 ^a^	0.212	0.487	12	0.277	0.384

%Brix: colostrum refractometric index; colostral IgG and foal serum IgG concentrations were determined by sandwich ELISA; ^a^ one SCFP foal died a few days after birth; ^b^ one CON colostrum sample was unusable; *n*: number; *p*: *p*-value; and *r*: *r*-value.

## Data Availability

None of the data were deposited in an official repository. The data that support the study findings are available upon reasonable request.

## Supplementary Materials

The following supporting information can be downloaded at: https://www.mdpi.com/article/10.3390/ani14172459/s1, Table S1: Average age and parity of mares; Table S2: Expected and occurred foaling months and group association; Table S3: Diarrhea classification; Table S4: Foal vaccination-induced changes of blood cell counts do not correlate with %Brix, colostral IgG content and day 2 foals’ serum IgG concentration.
