# Supplementary material for: Feeding a Saccharomyces cerevisiae Fermentation Product to Mares in Late Gestation Alters the Biological Activity of Colostrum
Source: Animals (Basel). 2024 Aug 24;14(17):2459. doi: 10.3390/ani14172459 (PMC11394450; doi:10.3390/ani14172459)
Supplement: Supplementary file 1 [file animals-14-02459-s001.zip › animals-3104668-supplementary.pdf]

Table S1. Average age and parity of mares.

	n	Mean Age (Min-Max)	Mean Parity (Min-Max)
**Group (SCFP)**	14	10 (4 - 18)	3.25 (0 - 7)
**Group (CON)**	12	11 (4 - 19)	3.58 (0 - 7)

Table S2. Expected and occurred foaling months and group association.

Month	SCFP (*n* = 14)		CON (*n* = 12)	
	expected	occurred	expected	occurred
Jan	0	0	2	2
Feb	3	2	2	2
Mar	4	4	2	2
Apr	1	3	3	3
May	2	0	1	1
Jun	4	5	2	2

Supplemented group (SCFP); Control group (CON).

Table S3. Diarrhea classification.

Score	Feces consistence
1	Watery
2	Thin mushy
3	Mushy pasty
4	Formed

Score 1 + 2 were classified as diarrhea.

Table S4. Foal vaccination-induced changes of blood cell counts do not correlate with %Brix, colostral IgG content, and day 2 foals' serum IgG concentration.

Blood parameter [a]	%Brix	Colostral IgG	Foal serum IgG Day 2
	p [b]	*p*	*p*
Leukocytes	0.644	0.416	0.263
Neutrophilic granulocytes	0.794	0.383	0.312
Lymphocytes	0.595	0.072	0.576
Monocytes	0.969	0.203	0.640
$CD4^+$	0.833	0.213	0.773
$CD8^+$	0.356	0.603	0.920
$CD21^+$	0.564	0.390	0.332
$MHCII^+/ CD21^-$	0.305	0.152	0.955

%Brix: colostrum refractometric index; colostral IgG (mg/mL) and foal serum IgG concentrations (mg/mL) were determined by sandwich ELISA; a) The early vaccination response was calculated as the difference (absolute values after vaccination minus absolute values before vaccination) for each blood parameter; b) *p*-values were detected using a mixed model with Brix or the IgG values as a continuous variable.
